# Recovery of the Decreased Phagocytic Function of Peripheral Monocytes and Neutrophil Granulocytes following Cytoreductive Surgery in Advanced Stage Epithelial Ovarian Cancer

**DOI:** 10.3390/medicina59091602

**Published:** 2023-09-05

**Authors:** Anna Rebeka Kovács, Luca Lukács, László Pál, Sándor Szűcs, Kincső Sára Kovács, Rudolf Lampé

**Affiliations:** 1 Department of Obstetrics and Gynecology, Faculty of Medicine, University of Debrecen, 98. Nagyerdei krt., 4032 Debrecen, Hungary; 2 Department of Public Health and Epidemiology, Faculty of Medicine, University of Debrecen, 26. Kassai út, 4028 Debrecen, Hungary

**Keywords:** ovarian cancer, phagocytosis, monocyte, neutrophil granulocyte

## Abstract

(1) Monocytes and neutrophil granulocytes are the phagocytic cells of the innate immune system, playing a crucial role in recognizing and eliminating tumor-transformed cells. Our objective was to assess the impact of advanced-stage epithelial ovarian cancer (EOC) and cytoreductive surgery on the phagocytic function of peripheral monocytes and neutrophil granulocytes. We aimed to compare the pre- and postoperative phagocytic function of these immune cells in EOC patients with healthy control women. Additionally, we aimed to examine the influence of surgery on phagocytic function by comparing pre- and postoperative samples from patients with benign gynecological tumors. (2) We examined peripheral blood samples from 20 patients with FIGO IIIC stage high-grade serous EOC and 16 patients with benign gynecological tumors as surgical controls, collected before and seven days after tumor removal surgery, and from 14 healthy women. After separation, the cells were incubated with Zymosan-A particles, and the phagocytic index (PI) was assessed using immunofluorescence microscopy. One-way ANOVA, the Kruskal–Wallis H-test, and the paired samples *t*-test were used for the statistical analysis of the data. A significance level of *p* < 0.05 was applied. (3) Peripheral monocytes and neutrophils from EOC patients exhibited significantly lower preoperative PI values compared to healthy controls (*p* < 0.001; *p* < 0.001, respectively). Following cytoreductive surgery, the PI values of immune cells in EOC patients significantly increased by the 7th postoperative day (*p* < 0.001; *p* < 0.001), reaching levels comparable to those of healthy controls (*p* = 0.700 and *p =* 0.991). In contrast, there was no significant disparity in the PI values of cells obtained from pre- and postoperative blood samples of surgical controls when compared to healthy women (monocytes: *p* = 0.361 and *p* = 0.303; neutrophils: *p* = 0.150 and *p =* 0.235). (4) EOC and/or its microenvironment may produce factors that reduce the phagocytic function of monocytes and neutrophils, and the production of these factors may be reduced or eliminated after tumor removal.

## 1. Introduction

Ovarian cancer is the third most common type of gynecological malignancy and the leading cause of death from gynecological cancer in women. It ranks as the fifth leading cause of cancer-related mortality in women, accounting for approximately 207,000 global fatalities in 2020 [1,2]. The elevated mortality rates stem in part from the disease’s propensity to remain asymptomatic during its initial stages, only manifesting symptoms during its advanced phases. Common symptoms in these advanced stages include abdominal distention, abdominal discomfort, weight loss, and fatigue. Urinary urgency or constipation may also emerge. In later stages, the condition can lead to the development of ascites and cachexia.

The International Federation of Gynecology and Obstetrics (FIGO) staging system classifies patients with ovarian cancer into stages I to IV based on tumor size and extent [3]. The primary treatment for ovarian cancer is cytoreductive (debulking) surgery, which means that less than 1 cm of tumor tissue remains after surgery [4]. After debulking surgery, current guidelines recommend platinum-based adjuvant chemotherapy (carboplatin or cisplatin), in combination with taxane compounds such as paclitaxel [3].

The pathogenesis of ovarian epithelial tumors remains largely unknown. Various theories exist concerning the etiology of serous epithelial ovarian cancer (EOC) [5,6]. Recent years have witnessed an emerging understanding of the significant influence of the immune system on the clinical course of the disease [7]. Presently, multiple studies underscore the interaction between the host’s immune system and the tumor [8]. In recent years, it has become evident in ovarian cancer, there are several associations between immune function and the clinical course of the disease [9].

During carcinogenesis, the progenitor cell must navigate a series of regulatory processes and physiological imbalances, which it accomplishes through various mechanisms. These mechanisms encompass autonomy from growth signals, insensitivity to growth inhibitory signals, unrestricted cell proliferation, suppressed apoptosis, and the capacity for vascularization, invasion, and metastasis [10]. In the context of ovarian cancer, it has been observed that tumor cells can induce various alterations in the tumor stroma, transforming it into a pathological niche known as the tumor microenvironment (TME). The TME arises from the interplay between tumor cells, normal cells, and immunosuppressive components (including diverse subtypes of tumor-infiltrating T and B lymphocytes, NK cells, tumor-associated macrophages (TAM), and myeloid-derived suppressor cells). These components directly contribute to tumor pathogenesis, suppressing anti-tumor immunity, and promoting tumor growth and progression through the production of cytokines and chemokines. The cytokines involved in immunosuppression include interleukin (IL-10, IL-6), transforming growth factor (TGF-β), vascular endothelial growth factor (VEGF), prostaglandin E-2, IL-35, IL-1β, IL-8, and metalloprotease enzymes. As a result, the TME creates an optimal environment for tumor cell proliferation and tumors may present clinically [11,12]. 

As a major branch of the body’s immune defenses, the innate immune system serves as the first line of non-specific defense against infection and malignant cell transformations [13]. As part of the innate immune system, neutrophils are the most abundant leukocytes in the bloodstream and serve as the frontline defense during instances of inflammation and infections [14,15]. The intrusion of microorganisms triggers an inflammatory reaction that summons neutrophils from circulation into the affected tissues, where neutrophils deploy an array of mechanisms—primarily phagocytosis, the release of antimicrobial substances, and the formation of neutrophil extracellular traps (NETs)—to eliminate the invading microorganisms. Neutrophils exhibit the capacity to produce numerous cytokines and chemokines, thereby influencing the inflammatory response [15]. Monocytes ranked as the third most abundant population of immune cells in peripheral blood, following neutrophils and lymphocytes. Within a day, the majority of monocytes exit circulation and migrate into tissues to replenish macrophages. Only a small fraction of monocytes remains in circulation for several days [16]. Monocytes and neutrophil granulocytes are the phagocytic cells of the innate immune system, playing a vital role in eliminating tumor-transformed cells and thereby offering defense against tumor development. This phagocytic process serves the purpose of eradicating microbes and apoptotic or tumor cells, and triggering the activation of adaptive immune cells through antigen presentation [13].

While a decline in the phagocytic function of peripheral monocytes and neutrophil granulocytes has been observed in various pathologies and diseases [17,18,19,20], the current literature on the phagocytic function of these cells in the context of ovarian cancer remains limited.

Therefore, our aim was to assess the impact of advanced-stage, high-grade serous EOC and cytoreductive surgery on the phagocytic function of peripheral monocytes and neutrophil granulocytes. This study aims to compare the pre- and postoperative phagocytic function of these immune cells in EOC patients with healthy control women. Additionally, the influence of surgery on phagocytic function will be examined by comparing pre- and postoperative samples from patients with benign gynecological tumors. By investigating these aspects, our objective is to enhance the understanding of ovarian cancer pathogenesis and its immunological implications.

## 2. Materials and Methods

With the permission of the Ethics Committee of the University of Debrecen and after obtaining informed consent, we analyzed blood samples from patients with Epithelial Ovarian Cancer (EOC) and patients with benign gynecologic tumors who underwent primary laparotomic surgery for tumor removal between 2017 and 2019 at the Obstetrics and Gynecology Clinic of the University of Debrecen Clinical Center.

For the EOC group, selection criteria included the histological type and FIGO stage of the disease. Our study specifically included patients with FIGO IIIC stage high-grade serous EOC. Cases with an inoperable status discovered during surgery or post-operative histopathology confirming a different histological type were excluded from the study. Another inclusion criterion was complete cytoreduction during primary surgery, resulting in the exclusion of patients with more than 1 cm of residual tumor tissue left after surgery. Additionally, we collected peripheral blood samples from healthy women without any history of cancer. The study participants had no history of diabetes, immunosuppressive diseases, or serious illnesses, and they were not taking regular medication.

Peripheral blood sampling was conducted using Vacutainer-type EDTA closed system blood collection tubes (Beckton-Dickinson, Cedex, France). Preoperative blood samples were obtained on the morning of the surgery day for all patients, while postoperative blood samples were taken on the 7th day after surgery.

The patients’ mononuclear cells and granulocytes were isolated using a method previously described [21]. Peripheral blood samples were mixed with equal volumes of Hanks’ solution (pH 7.4) and layered onto a discontinuous Ficoll gradient (1.077 and 1.119 g/cm^3^). Centrifugation was performed at 400 *g* for 30 min at 20 °C. Mononuclear and polymorphonuclear cells were collected from the top and interface of the Ficoll gradients. Subsequently, the cells were washed twice with Hanks’ solution. Their viability was assessed using the trypan blue exclusion test and found to be between 96% and 98%. The purity of the granulocyte suspensions ranged from 95% to 98%, based on morphological evaluation.

Labeling of zymosan particles with fluorescein isothiocyanate (FITC) was conducted following a method previously described in the literature [22]. Zymosan-A particles (10^8^/cm^3^) were incubated at 37 °C for 60 min in carbonate buffer (pH 9.6) containing 0.01 mg/cm^3^ of FITC. Subsequently, they underwent three washes and were opsonized in Hanks’ solution containing 50% human AB serum at 37 °C for 30 min. The labeled and opsonized particles (FITC-OZ) underwent three additional washes and were then stored at −20 °C in Hanks’ solution (3 × 10^7^/cm^3^) until the phagocytosis assay.

In the phagocytosis assay, phagocytosis of FITC-OZ was determined as described previously [23]. In brief, 10^6^ granulocytes and mononuclear cells were placed in 300 µL aliquots of Hanks’ solution containing 5% heat-inactivated human AB serum onto chamber slides. This allowed the cells to adhere for 30 min at room temperature. Subsequently, non-adherent cells were removed through washing with Hanks’ solution. FITC-OZ particles were introduced as phagocytosis targets. Adherent cells and FITC-OZ particles (3 × 10^6^/well) were incubated in 300 µL aliquots of Hanks’ solution at 37 °C in a 5% CO_2_: 95% humidified air environment for 60 min.

Following the incubation, trypan blue solution was used to quench the fluorescence of non-ingested zymosan particles [24]. After dissecting the chambers, monocytes and granulocytes were fixed on the plates using a 4% paraformaldehyde solution for 30 min.

Monocytes were identified using an indirect immunofluorescent method. The cells were first labeled with an anti-CD14 monoclonal antibody and then with immunoglobulin G conjugated to Dylight 594 fluorescent dye. The nuclei of both granulocytes and monocytes were stained with 4,6-diamidino-2-phenylindole (DAPI). 

**Figure 1:**
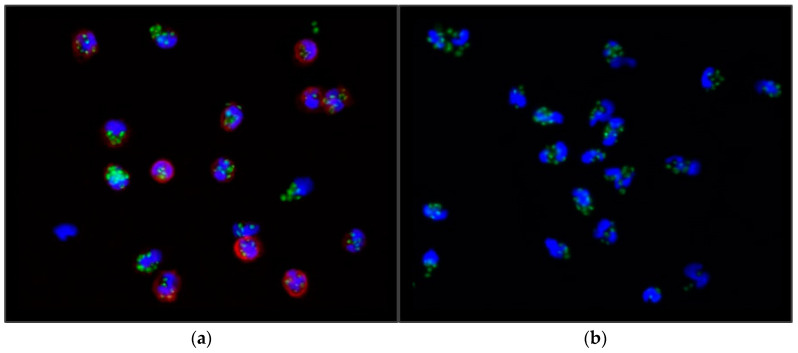
Fluorescent microscopy images of the (**a**) monocytes and (**b**) neutrophil granulocytes. Green: phagocytosed Zymosan particles; blue: nuclei of the cells stained with DAPI; red: stained cell membranes of the monocytes.

The count of FITC-OZ particles per cell was determined using an Axioplan fluorescent microscope (Zeiss Oberkochen, Germany). Cells from randomly selected microscopic fields were examined (see Figure 1), and subsequently, the phagocytic index (PI) was calculated by dividing the number of Zymosan-A particles phagocytosed by 100 cells by 100 (1). Hence, the PI signifies the mean number of zymosan particles phagocytosed per individual cell.

(1) $$\mathrm{PI}=\frac{number\;of\;phagocytozed\;particles\;by\;100\;cells}{100}$$

Statistical calculations were conducted using SPSS 25.0. The normality of data distribution was assessed using the Kolmogorov–Smirnov and Shapiro–Wilk tests. For data with a normal distribution, one-way ANOVA (analysis of variance) was utilized to compare PIs among different groups and to analyze clinical data of study subjects. In cases of non-normal distribution, the non-parametric Kruskal–Wallis H-test was employed. Preoperative and postoperative PIs were compared using the Student’s paired sample *t*-test. A significance level of *p* < 0.05 was considered statistically significant.

## 3. Results

### 3.1. Clinical Data of Subjects

The clinical data of the patients in the study are presented in Table 1. Throughout the study period, a total of *n* = 20 patients with IIIC stage high-grade serous epithelial ovarian cancer underwent complete primary cytoreductive surgery (with less than 1 cm of tumor tissue remaining after surgery, as described by Schorge et al., 2018 [4]). According to our previously described patient selection criteria, a total of five patients were excluded from the study group due to incomplete cytoreduction surgery (*n* = 3), different FIGO stage (*n* = 1; FIGO IV stage disease), or histological type (*n* = 1; mucinous EOC).

Within the surgical control group (total *n* = 16), 14 patients underwent surgery for uterine fibroids, and 2 patients underwent surgery for benign ovarian cysts. 

The healthy control group consisted of *n* = 14 healthy control women.

The Kolmogorov–Smirnov and Shapiro–Wilk tests revealed that the age and BMI values of the patients, as well as the PIs of the monocytes and neutrophil granulocytes, followed a normal distribution. However, the gravidity and parity values exhibited deviations from the normal distribution.

As indicated in Table 1, there were no significant differences in age (*p* = 0.077), gravidity (*p* = 0.109), parity (*p* = 0.514), and body mass index (*p* = 0.378) at the time of blood sampling among EOC patients, healthy women, and surgical controls.

### 3.2. Phagocytic Function of Monocytes

**Figure 2:**
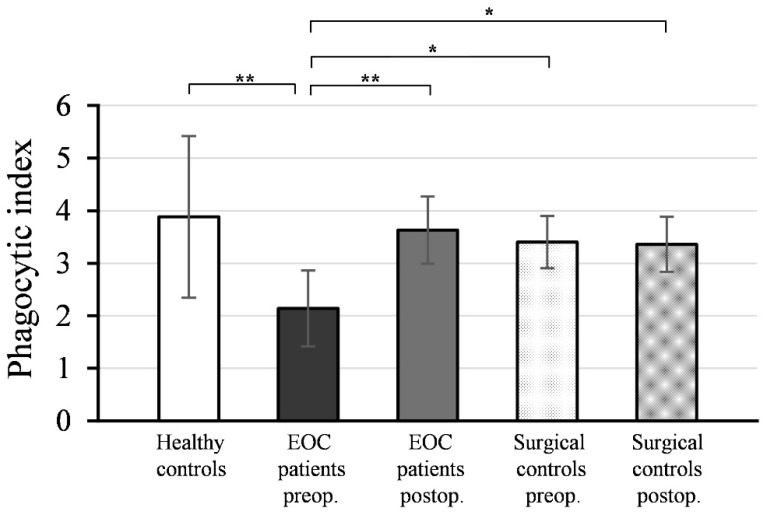
Phagocytic indices (PI) of monocytes from patients with epithelial ovarian cancer (EOC) (*n* = 20), surgical controls with benign gynecological tumors (*n* = 16), and healthy controls (*n* = 14). Preoperative blood sampling was performed on the morning of the tumor removal surgery day for all patients, and postoperative blood sampling was conducted on the 7th day after surgery. Mean values ± SD are presented. ** *p* < 0.001 denotes a significant difference between preoperative and postoperative PI values of monocytes in EOC patients, and between preoperative PI values of monocytes in EOC patients and healthy controls. * *p* < 0.01 indicates significant differences between preoperative PI values of monocytes in EOC patients and preoperative/postoperative PI values of surgical controls.

Figure 2 illustrates the mean values and standard deviations (± SD) of the phagocytic indices (PI) of monocytes isolated from peripheral blood samples collected pre- and postoperatively from EOC patients, surgical controls, and healthy women. 

As depicted, the preoperative PI values of monocytes from EOC patients (PI = 2.14 ± 0.72) were significantly lower compared to healthy controls (PI = 3.88 ± 1.54; *p* < 0.001). Postoperative PI values of monocytes from EOC patients (PI = 3.63 ± 0.64) exhibited a significant increase (*p* < 0.001) compared to preoperative values and reached a level comparable to healthy controls (*p* = 0.700).

There was no statistically significant difference between the PI values of monocytes isolated from preoperative (PI = 3.40 ± 0.50) and postoperative (PI = 3.36 ± 0.52) blood samples in the surgical control group (*p* = 0.567), and these values were not significantly different from the PI values of monocytes from healthy controls (preoperative: *p* = 0.361; postoperative: *p* = 0.303).

The preoperative PI values of monocytes in EOC patients were significantly lower compared to both the preoperative (*p* < 0.01) and postoperative PI values of the surgical controls (*p* < 0.01).

### 3.3. Phagocytic Function of Neutrophil Granulocytes

**Figure 3:**
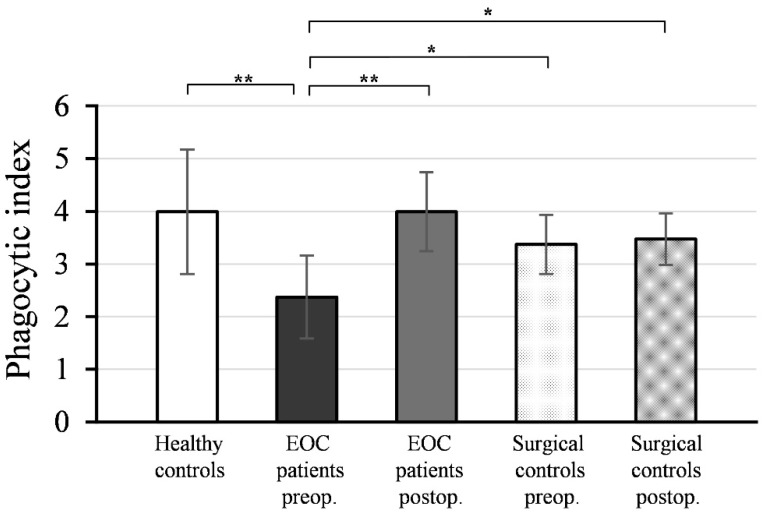
Phagocytic indices (PI) of neutrophil granulocytes from patients with epithelial ovarian cancer (EOC) (*n* = 20), surgical controls with benign gynecological tumors (*n* = 16), and healthy controls (*n* = 14). Preoperative blood sampling was performed on the morning of the tumor removal surgery day for all patients, and postoperative blood sampling was conducted on the 7th day after surgery. Mean values ± SD are presented. ** *p* < 0.001 denotes a significant difference between preoperative and postoperative PI values of neutrophils in EOC patients, and between preoperative PI values of monocytes in EOC patients and healthy controls. * *p* < 0.01 indicates significant differences between preoperative PI values of neutrophils in EOC patients and preoperative/postoperative PI values of surgical controls.

Figure 3 shows the mean values and standard deviations (±SD) of the phagocytic indices (PI) of neutrophil granulocytes isolated from peripheral blood samples collected pre- and postoperatively from EOC patients and surgical controls, as well as healthy women. 

As depicted, the preoperative PI values of neutrophil granulocytes from EOC patients (PI = 2.37 ± 0.79) were significantly lower compared to healthy controls (PI = 3.99 ± 1.18; *p* < 0.001). Postoperative PI values of neutrophils from EOC patients (PI = 3.99 ± 0.75) exhibited a significant increase (*p* < 0.001) compared to preoperative values and reached a level comparable to healthy controls (*p* = 0.991).

There was no statistically significant difference between the PI values of neutrophil granulocytes isolated from preoperative (PI = 3.37 ± 0.56) and postoperative (PI = 3.47 ± 0.49) blood samples in the surgical control group (*p* = 0.542), and these values were not significantly different from the PI values of neutrophils from healthy controls (preoperative: *p* = 0.150; postoperative: *p* = 0.235). 

The preoperative PI values of neutrophil granulocytes in EOC patients were significantly lower compared to both the preoperative (*p* < 0.01) and postoperative PI values of the surgical controls (*p* < 0.01).

## 4. Discussion

Ovarian cancer is the leading cause of cancer death worldwide [25], with around 314,000 new cases diagnosed in 2020 [1]. The most prevalent subtype is EOC [25], which is usually diagnosed at an advanced (FIGO III-IV) stage, when five-year overall survival is as low as 25% [26]. The most common histological type of EOC is high-grade serous ovarian cancer (75% of all EOC patients) [3].

In recent years, extensive research has focused on understanding the intricate interactions between ovarian cancer and the immune system [7]. 

Phagocytosis is a multistep cellular process involving the recognition of target cells, cellular engulfment, and subsequent lysosomal digestion, all of which are meticulously orchestrated by receptor–ligand interactions between phagocytes and their intended targets [13]. 

In this study, we examined the phagocytic function of peripheral monocytes and neutrophil granulocytes using blood samples collected before and after cytoreductive surgery in patients with advanced-stage, high-grade serous epithelial ovarian cancer. Our findings demonstrated a significant reduction in the preoperative phagocytic function of peripheral monocytes and neutrophil granulocytes from EOC patients compared to the corresponding cells from healthy controls. 

Similar findings were reported in a previous study, where the phagocytic function of monocytes isolated from peripheral blood samples of breast cancer patients showed a significant reduction in relation to disease severity, compared to the phagocytic activity of monocytes from healthy controls [27]. Based on the above data, it is conceivable that there could be a correlation between the decreased phagocytic function of monocytes and/or neutrophil granulocytes and the severity of ovarian carcinoma. However, our research did not allow for a stage-, differentiation degree-, or histological group-dependent comparison of the impact of phagocytosis on ovarian cancer. Therefore, determining this requires further investigation. While a decline in the phagocytic function of peripheral monocytes and neutrophil granulocytes has been observed in various pathologies and diseases, such as endometriosis before surgery [18,19], the current literature on the phagocytic function of these cells in the context of ovarian cancer remains limited. 

In our study, the PI values of monocytes and neutrophil granulocytes in EOC patients were significantly increased at postoperative measurement compared to preoperative values and reached the PI levels of the corresponding cells in healthy controls. While investigating the phagocytic function of peripheral monocytes in breast cancer patients, a study by Arsenijević et al. (2005) revealed a reduction in monocyte phagocytic function following surgical therapy [27], in contrast to the increased phagocytic function we observed. Notably, during the course of chemotherapy, there was a further decline in the phagocytic index, followed by a recovery three months after the final chemotherapy cycle. However, even at that point, the phagocytic index remained notably lower than that of monocytes from healthy control individuals [27]. Based on our results, we hypothesize that the increase in phagocytic function after cytoreductive surgery in ovarian cancer patients is due to the removal of ovarian cancer tissue, which may indicate an impact of the tumor on the immune system.

While healthy and normal tissues possess inherent mechanisms to evade self-elimination by phagocytes, often through the expression of anti-phagocytosis molecules referred to as ‘don’t eat me’ signals, cancer cells depend even more on similar mechanisms to evade immune-mediated eradication. Tumor cells heavily rely on the expression of ‘don’t eat me’ signals, which encompass molecules such as CD47, programmed cell death 1 ligand 1, β2-microglobulin, and other yet unidentified ligands. By binding to receptors on phagocytes, these molecules successfully obstruct the phagocytosis of tumor cells [13].

One of the most frequently observed phenotypic changes induced by cancer in peripheral blood monocytes is the acquisition of immunosuppressive activity. Recent studies have underscored the diagnostic, predictive, and prognostic potential of these phenotypic shifts in peripheral blood monocytes. The ease of obtaining monocytes through blood sampling presents a promising avenue for clinical oncology. The emergence of immunosuppressive monocytes in the bloodstream of cancer patients, coupled with their extensively documented connection to unfavorable prognosis, strongly implies that the reprogramming of monocytes induced by cancer plays a pivotal role in driving tumor progression [16]. Research has demonstrated that neutrophil granulocytes isolated from peripheral blood samples of ovarian cancer patients exhibit a proinflammatory phenotype. These cells show an increased production of reactive oxygen species and elevated expression of adhesion molecules when compared to blood samples from healthy control individuals [28]. These data can support the findings of recent studies that have indicated that calculated ratios of white blood cells, such as the neutrophil-to-lymphocyte ratio (NLR), monocyte-to-lymphocyte ratio (MLR), and platelet-to-lymphocyte ratio (PLR), derived from pre-treatment blood samples of patients, hold prognostic value for ovarian cancer [29,30,31,32,33]. Elevated NLR in peripheral blood samples collected before therapy has been observed to correlate with worse prognoses in ovarian cancer patients [34]. These inflammatory biomarkers, determined through complete blood cell counts, have also been evaluated for their predictive value in therapy selection upon ovarian cancer diagnosis [29]. Moreover, MLR and Cancer antigen-125 have been identified as significant, independent predictors of the extent of tumor reduction achievable during primary debulking surgery in ovarian cancer [35]. 

To ascertain the potential impact of surgery on phagocyte function, we additionally assessed the phagocyte function of monocytes and neutrophil granulocytes isolated from both pre- and postoperative blood samples of patients who had undergone abdominal surgery for benign gynecological tumors (surgical control group). In the surgical controls, the PI values of monocytes and neutrophils from preoperative blood samples did not exhibit significant differences from the postoperative PI values. Moreover, no significant difference was observed in the phagocytic function of preoperative monocytes and neutrophil granulocytes among patients with benign gynecological tumors (surgical control group) and healthy control women. Numerous studies have explored the impact of various surgical procedures on immune cell function, with a subset specifically examining the phagocytic function of neutrophil granulocytes and/or monocytes. The prevailing trend observed in many of these investigations indicates that the phagocytic function of cells isolated during or after surgery tends to decrease or remain stable compared to the preoperative phase [36,37,38]. This alignment with our findings underscores the consistency in results across these studies.

Our study has certain limitations, including the relatively small patient cohort and the absence of the identification of potential plasma factors. Enhancing the robustness of our findings could be achieved by expanding the sample size through a multi-center study and incorporating additional methodologies to pinpoint plasma factors.

## 5. Conclusions

In this study, we found that the preoperative phagocytic function of peripheral monocytes and neutrophil granulocytes was reduced in patients with advanced-stage, high-grade serous EOC compared to healthy controls. Remarkably, postoperative measurements exhibited a significant increase in phagocytic function, bringing these values to levels comparable to those of healthy controls. 

Given that there was no significant difference between the pre- and postoperative phagocytic function of monocytes and neutrophil granulocytes in the surgical control group (and no difference compared to healthy controls), it is reasonable to infer that the observed changes in phagocytic function among EOC patients are not a direct result of the surgical intervention, but rather are specific to EOC.

Based on our findings, the observed elevation in phagocytic function subsequent to cytoreductive surgery implies that EOC tissue and/or its TME could potentially generate factors that hinder the phagocytic function of peripheral neutrophil granulocytes and monocytes. Moreover, our results suggest that the production of these factors may be diminished or eliminated following the complete surgical removal of the tumor.

## Figures and Tables

**Table 1:** Clinical data of the patients with epithelial ovarian cancer (EOC), patients with benign gynecological tumor (surgical control), and healthy control women participating in the study.

Clinical Data	EOC Patients (*n* = 20)	Surgical Controls (*n* = 16)	Healthy Controls (*n* = 14)	*p* Value
Age [year] ^1^	62.25 ± 10.80	55.00 ± 12.44	53.43 ± 13.18	NS (*p* = 0.077)
Gravidity ^2^	2 (0–5)	1 (0–3)	2.5 (0–9)	NS (*p* = 0.109)
Parity ^2^	1 (0–3)	1 (0–2)	2 (0–3)	NS (*p* = 0.514)
Body mass index [kg/m^2^] ^1^	26.21 ± 3.73	25.42 ± 4.13	27.50 ± 4.39	NS (*p* = 0.378)
Tumor type	High-grade serous EOC (*n* = 20)	Uterine myoma (*n* = 14) ovarian cyst (*n* = 2)		
Tumor stage (FIGO) ^3^	IIIC ^3^	NA		
Optimal tumor reduction ^4^	100%	NA		

NA: not applicable. NS: not significant. ^1^ Mean values ± standard deviations are presented. ^2^ Values are expressed as median (range). ^3^ FIGO (International Federation of Gynecology and Obstetrics): a staging system for ovarian cancer that allows patients to be classified as stage I-IV based on the size and extent of the tumor. IIIC: tumor involving 1 or both ovaries, with cytologically or histologically confirmed spread to the peritoneum outside the pelvis (>2 cm) and/or metastasis to regional lymph nodes.^4^ Optimal tumor reduction: during the tumor removal surgery <1 cm tumor tissue remains.

## Data Availability

The data presented in this study are available by contacting the corresponding author.
